# Effects of Elevated Aluminum Concentration and Distribution on Root Damage, Cell Wall Polysaccharides, and Nutrient Uptake in Different Tolerant *Eucalyptus* Clones

**DOI:** 10.3390/ijms232113438

**Published:** 2022-11-03

**Authors:** Wannian Li, Saif Ullah, Yuanyuan Xu, Tiandao Bai, Shaoming Ye, Weixin Jiang, Mei Yang

**Affiliations:** 1 Guangxi Key Laboratory of Forest Ecology and Conservation, Forestry College, Guangxi University, Nanning 530004, China; 2 Key Laboratory of National Forestry and Grassland Administration on Cultivation of Fast-Growing Timber in Central South China, Forestry College, Guangxi University, Nanning 530004, China

**Keywords:** *Eucalyptus*, aluminum stress, resistant physiology, root tip, subcellular component

## Abstract

Aluminized acidic soil can damage *Eucalyptus* roots and limit tree growth, hindering the productivity of *Eucalyptus* plantations. At present, the negative impacts of elevated aluminum (Al) on the cell morphology and cell wall properties of *Eucalyptus* root tip are still unclear. In order to investigate the responses of two different tolerant clones, *Eucalyptus urophylla* (G4) and *Eucalyptus grandis* × *Eucalyptus urophylla* (G9), to Al toxicity, seedling roots were treated hydroponically with an Al solution, and the polysaccharide content in the root tip cell wall and the characteristics of programmed cell death were studied. The results show that the distribution of Al was similar in both clones, although G9 was found to be more tolerant to Al toxicity than G4. The Al^3+^ uptake of pectin in root tip cell walls was significantly higher in G4 than in G9. The root tip in G4 was obviously damaged, enlarged, thickened, and shorter; the root crown cells were cracked and fluffy; and the cell elongation area was squeezed. The lower cell wall polysaccharide content and PME activity may result in fewer carboxylic groups in the root tip cell wall to serve as Al-binding sites, which may explain the stronger Al resistance of G9 than G4. The uptake of nitrogen and potassium in G4 was significantly reduced after aluminum application and was lower than in G9. Al-resistant *Eucalyptus* clones may have synergistic pleiotropic effects in resisting high aluminum–low phosphorus stress, and maintaining higher nitrogen and potassium levels in roots may be an important mechanism for effectively alleviating Al toxicity.

## 1. Introduction

*Eucalyptus*, a typical fast-growing hardwood that is widely planted in tropical and subtropical regions, has an important impact on the world timber market due to the high lumber yield. More than 22.57 million hectares of *Eucalyptus* have been planted in 95 countries [1]. *Eucalyptus* plank production has an important impact on the world timber market; thus, the development of *Eucalyptus* plantations is important in supporting the market. The annual output of fast-growing *Eucalyptus* exceeds one-third of China’s total timber output. The *Eucalyptus* industry has formed a complete industrial chain, including the production of seedlings, fertilizer, wood, pulp and paper, wood-based panels, biomass energy, and forest by-products. *Eucalyptus* naturally adapts to various environmental conditions, but plantations have been mainly established in acidic soils [2]. In acidic soils (pH < 5), Al^3+^ is more soluble; thus, aluminum (Al) mainly exists in the form of exchangeable Al, which is then absorbed and accumulated by the roots [3]. The primary toxic effect of Al is inhibition of root elongation, causing severe damage to the root system [4,5]. Dissolved Al^3+^ in acidic soils has been regarded as a potential factor limiting tree growth and development [6]. It has been reported that various *Eucalyptus* species or clones demonstrate different responses under Al exposure [7]. Soil acidity significantly affects *Eucalyptus* forest soil, causing the accumulation of Al in roots, although the productivity of *Eucalyptus* is not as highly affected as other crops by increased soil acidity [3]. It was reported that root elongation of *E. camaldulensis* was not affected after 20 days of treatment with 1 mM Al [8].

A positive effect on plant growth of Al at low concentrations has often been observed in *Eucalyptus* species. Some species demonstrate strong Al tolerance, which can be detected by increased chlorophyll a, net photosynthetic rate, and water use efficiency; minor production of reactive oxygen species and reduced alterations in stress indicators; and the production of Al-binding ligands in the roots [9,10]. *E. grandis* has also been proved to be more tolerant to Al than *E. platyphylla* [11], based on changes in micronutrient concentration, photosynthetic pigments, gas exchange, and morphological indicators. Disorders in *Eucalyptus* correlated with growth, physiology, and nutrients resulting from Al toxicity have been described in the literature [12,13]. Studies have also shown that the functions and processes associated with cellular disruption are also inhibited by Al stress [14,15]. Al was shown to increase the amount of pectin, hemicellulose, and cellulose in *Eucalyptus* tip cell walls [16]. Pectin methylesterase catalyzes the demethylation–esterification of cell wall polygalacturonic acid, which is involved in important developmental processes in plants, including cell elongation [17]. When the pectin content of root tip cells is high, plants are more sensitive to Al toxicity, and the combination of Al and cell wall pectin is important in the expression of Al toxicity and resistance [18]. Fluctuations in polysaccharide content in apical cell walls can reduce water and nutrient uptake in roots and cell wall ductility [16] as well as decrease the plasticity of cell walls and inhibit the growth of plant roots [19,20]. Programmed cell death (PCD) induced by Al is considered an important reason for Al phytotoxicity [21]. Root border cells (RBCs) develop from root cap cells [22]. The protective effect of RBCs and their associated mucus represents an external detoxification mechanism in plants [23].

Plants have evolved various levels of tolerance to Al toxicity. Therefore, cultivating Al-tolerant plants is an effective way to sustainably and efficiently detoxify acidic soils containing Al [24]. Silviculture practices require trees that not only have high yield but also good environmental adaptability [25]. The studies on the response of *Eucalyptus* to Al reported above mainly focused on different *Eucalyptus* species. At present, *Eucalyptus* trees have entered the stage of clonal management, and elite hybrid clones have been cultivated and planted. Using fast-growing *Eucalyptus* hybrid clones to study Al toxicity and its regulation mechanism will help in finding more precise Al response genes in *Eucalyptus*. In our previous study, the Al tolerance of four fast-growing *Eucalyptus* clones in southern China was graded as follows: *Eucalyptus grandis* × *Eucalyptus urophylla* (G9) was more tolerant, followed by *Eucalyptus grandis* × *Eucalyptus urophylla* (G12) and *Eucalyptus urophylla* × *Eucalyptus camaldulensis* (G3), and *Eucalyptus urophylla* (G4) was the most susceptible [26,27]. Therefore, we hypothesized that the difference in Al tolerance between two different *Eucalyptus* clones inhibited by the distribution of Al^3+^ within the root tip may represent different responses through root tip cell morphology, the regulation of polysaccharide content within the subcellular components of the root tip, and root nutrient uptake. Consequently, our initial study aim was to reveal the cellular response mechanisms involved in Al toxicity resistance among fast-growing *Eucalyptus* clones with different Al-resistant properties, in order to provide a reference for the breeding of excellent Al-resistant clones.

## 2. Results

### 2.1. Effect of Al on Root Tip Surface, PCD Detection, and Change in Root Tip Length

**Figure 1:**
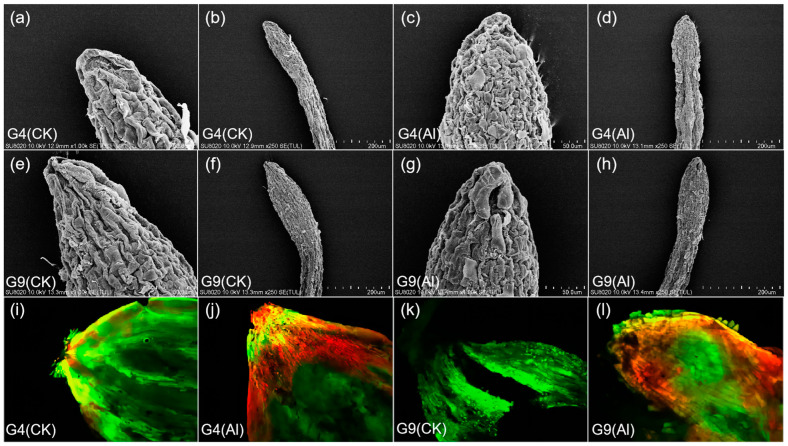
SEM photograph and PCD of root apex of *Eucalyptus urophylla* (G4) and *Eucalyptus grandis* × *Eucalyptus urophylla* (G9) after 24 h of Al stress at different magnifications. (**a**,**b**) Al-sensitive G4 samples without Al treatment; (**c**,**d**) G4 samples with Al treatment; (**e**,**f**) Al-resistant G9 samples without Al treatment; (**g**,**h**) G9 samples with Al treatment; (**i**–**l**) root tip cells stained with FDA-PI and observed under a fluorescence microscope, where green fluorescence indicates viable cells and red fluorescence indicates dead cells. (**a**,**c**,**e**,**g**,**i**–**l**) 1000× magnification; (**b**,**d**,**f**,**h**) 250× magnification. SEM, scanning electron microscopy, PCD, programmed cell death, FDA-PI, fluorescein diacetate–propidium iodide.

Under Al stress, root tip cells became swollen, thicker, and shorter (Figure 1). Damage to the root tip surface in G4 was the most obvious. Root cap cells were damaged, and the root tip epidermis was cracked, fluffy, rough, and tightly ravine-like (Figure 1c,d). In contrast, G9 root tip cells under Al stress showed a concave surface but were much looser with less cracking (Figure 1g,h). In the control treatments, the root tip cell surfaces were neat, with shallow grooves, minimal cracking, and greater spacing between cells in the elongation zone (Figure 1a,b,e,f). However, after Al induction, the cell walls were wrinkled and the adjacent cells became closer and smaller, forming a cellular array (Figure 1c,d,g,h). To determine if Al stress is related to apoptosis, fluorescence detection using fluorescein diacetate–propidium iodide (FDA-PI) staining was performed. As shown in Figure 1i–l, Al-induced cells displayed intense red or orange colors, indicating apoptosis in G4, while the control only showed some light pink, indicating that the majority of cells were still active. The root apical area of Al-tolerant G9 also showed evidence of apoptosis, but much less than G4. The control specimen only fluoresced bright green, indicating normal growth.

**Figure 2:**
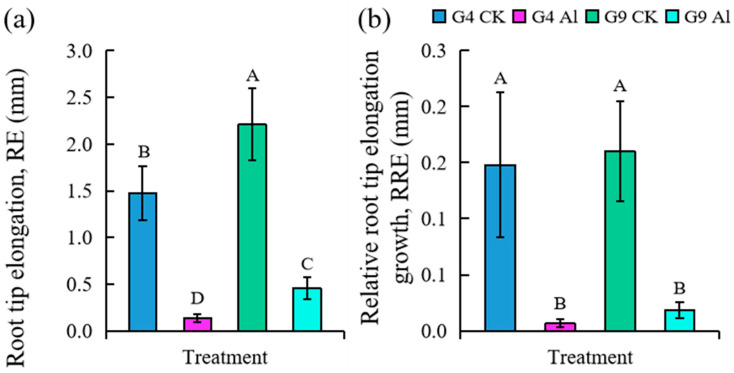
Effects of Al stress on (**a**) root tip elongation (RE) and (**b**) relative root tip elongation (REE) of *Eucalyptus* clones G4 and G9 after 24 h of Al treatment. Note: data in histogram represent root length after 24 h of Al treatment minus root length at beginning of Al treatment. Bars with different letters indicate significant difference at *p* < 0.05; three replicated samples used (*n* = 3).

The effect of Al stress on the root elongation growth of two different *Eucalyptus* clones is shown in Figure 2. It was obvious that root elongation in G4 and G9 was significantly inhibited after exposure to Al (*p* < 0.05), and significantly less inhibition was seen in G9 than G4 (Figure 2a). The ratio of root length growth after Al treatment to the initial value showed a similar trend (Figure 2b).

### 2.2. Distribution of Al^3+^ in Subcellular Fractions of Root Tip Cells

**Figure 3:**
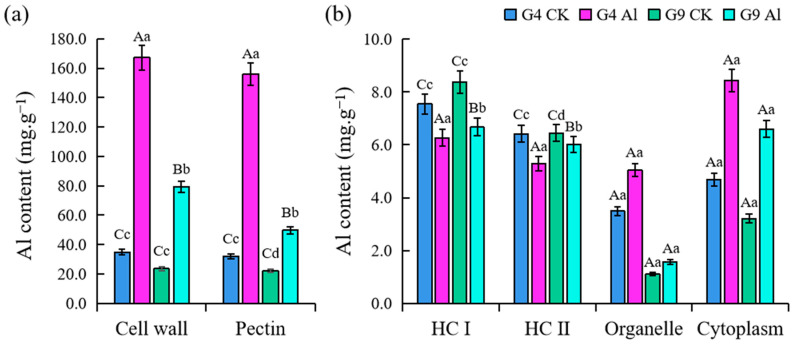
Changes in micro-Al content of root tip cell walls of *Eucalyptus* clones G4 and G9 after 24 h of Al stress. CK indicates control treatment (0 mM Al^3+^), and Al indicates Al treatment (4.4 mM Al^3+^). Cell walls from 1 cm root sections were extracted in the experiment. Note: different lowercase and uppercase letters on vertical bars indicate significant differences at the levels of 0.05 and 0.01, respectively. (**a**) Al content in cell wall and pectin; (**b**) Al content in subcellular fractions.

The concentration of Al^3+^ was significantly higher in G4 and G9 plants (*p* < 0.01) compared to the control, except hemicellulose I (HC I) and hemicellulose II (HC II) (Figure 3). Prior to Al^3+^ treatment, there was no significant difference in Al^3+^ concentration between G4 and G9; however, the level of Al was much higher in G4 than G9 after 24 h. The Al^3+^ concentration of the pectin in G4 and G9 was 3.9 and 2.3 times higher, respectively, than in the control plants, while the Al^3+^ uptake by pectin accounted for 93.3% in G4 and 62.7% in G9. The amount of Al^3+^ in the organelles and extracellular matrix in the Al-sensitive clone G4 was 1.4 and 1.8 times higher, respectively, compared to the control. The amount of Al^3+^ in the organelles and extracellular matrix in G9 was 1.4 and 2.05 times higher, respectively, than in the control plants.

### 2.3. Contents of Galacturonic Acid (GA) and Total Sugar in Subcellular Components

**Figure 4:**
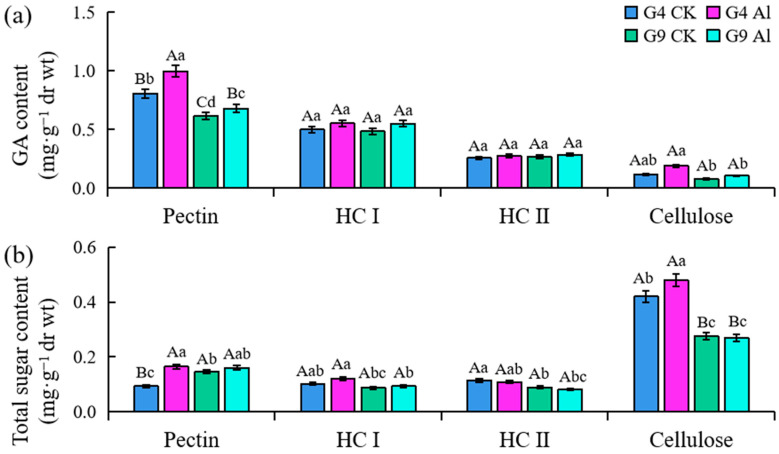
(**a**) Galacturonic acid (GA) and (**b**) total sugar content of cell walls extracted from root tips of *Eucalyptus* clones G4 and G9. Two-month-old seedlings were exposed to 0 or 4.4 mM Al solution for 24 h. Root apices were cut, and cell wall polysaccharides were fractionated into pectin, hemicellulose I (HC I), and hemicellulose II (HC II) for GA content measurement. Note: different lowercase and uppercase letters indicate significant differences at the levels of 0.05 and 0.01, respectively.

The GA content of pectin was significantly greater than that of the control group (*p* < 0.01) following Al exposure (Figure 4a). The increased GA content was significantly higher (23.7%) in Al-sensitive G4 than Al-resistant G9. The GA in cellulose tended to increase under Al stress. No significant difference was detected in the amount of HC I or II in either *Eucalyptus* clone after exposure to Al. Similarly, the total sugar in the principal components of the cell walls increased significantly in G4, which is more sensitive than G9. As shown in Figure 4b, the total sugar content of the pectin, which was significantly different with and without Al treatment in the *Eucalyptus* clone G4 (*p* < 0.01), was 76.7% higher compared to the 0 mM Al^3+^ group in G4. Compared with the control group, after Al treatment, the total sugar content of pectin in the two clones increased significantly, although the growth rate of G4 was 7.36 times that of G9. The total sugar content in HC I of the two clones increased after Al treatment, but that in HC II was decreased.

### 2.4. PME Activity in Root Tip and Root Uptake of N, P, and K

**Figure 5:**
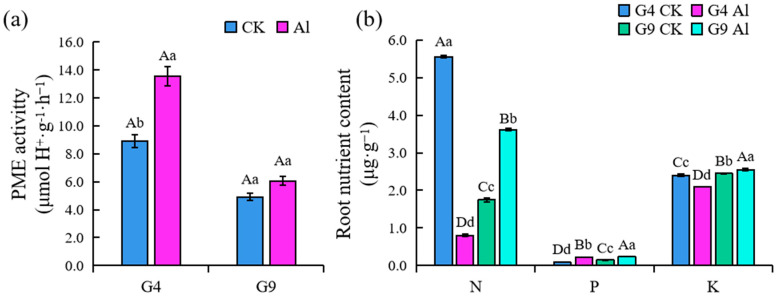
(**a**) PME activity in root tips and (**b**) N, P, and K content in roots of *Eucalyptus* clones G4 and G9. PME activity was determined colorimetrically. Note: different lowercase and uppercase letters indicate significant differences at the levels of 0.05 and 0.01, respectively. PME, pectin methylesterase; N, nitrogen; P, phosphorus; K, potassium.

Al treatments resulted in increased pectin methylesterase (PME) activity in both clones, as shown in Figure 5. PME activity was significantly higher in Al-sensitive G4 in the absence of Al (*p* < 0.05), while no significant difference was observed for G9 with or without Al. The PME activity of G4 root tips induced by Al stress was greater and accelerating pectin demethylation was easier. This allowed Al^3+^ to accumulate in the pectin of cell walls and further expanded the destructive effects of Al toxicity. After Al treatment, the nitrogen (N), phosphorus (P), and potassium (K) content in the roots of G4 and G9 fluctuated significantly (*p* < 0.01) (Figure 5b). Al toxicity greatly limited the absorption of N and K by G4 roots; compared with the controls, absorption of N and K significantly decreased by 85.6 and 12.6%, respectively. However, the accumulation of N and K in roots of G9 was not inhibited, but was significantly increased by 107.0 and 4.2%. The absorption of P increased significantly in both clones when stimulated by Al, but overall, G9 took up more P nutrients. The roots of G9 still maintained high Al tolerance and nutrient acquisition ability under Al stress.

### 2.5. Correlation Analysis of Al^3+^ Concentration with N, P, and K Content in Roots, GA and TS Content in Subcellular Fractions, and PME Activity

**Figure 6:**
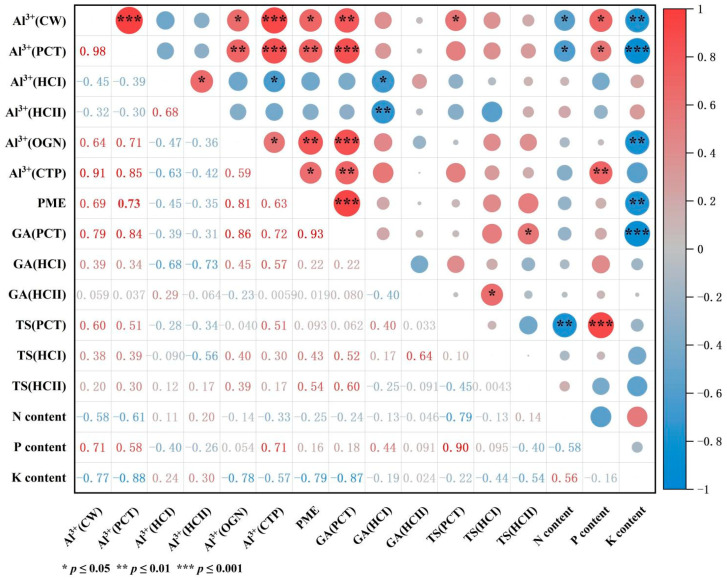
Interaction between Al^3+^ concentration in root tip subcellular components and PME activity, and GA, TS, and N, P, and K content under Al stress. Note: data of *Eucalyptus* clones G4 and G9 are combined to show correlations between Al^3+^ concentration and other indicators. Numbers in blocks represent correlation coefficient; 1 indicates completely positive correlation, −1 indicates completely negative correlation, and 0 indicates no correlation. Al^3+^(CW): Al^3+^ concentration of cell wall; Al^3+^(PCT): Al^3+^ concentration of pectin; Al^3+^(HC I): Al^3+^ concentration of hemicellulose I; Al^3+^(HC II): Al^3+^ concentration of hemicellulose II; Al^3+^(OGN): Al^3+^ concentration of organelle; Al^3+^(CTP): Al^3+^ concentration of cytoplasm. PME, pectin methylesterase; TS, total sugar content; N, nitrogen; P, phosphorus; K, potassium.

After Al treatment, the Al^3+^ content of cell walls was positively correlated with the Al^3+^ content of pectin and cytoplasm and the GA content of pectin (*p* ≤ 0.01), and the correlation coefficients were 0.98, 0.91, and 0.79, respectively (Figure 6). The Al^3+^ content of cell walls was positively correlated with the Al^3+^ content of organelles, PME activity, and the total sugar content of pectin (*p* ≤ 0.05). The Al^3+^ content of HC I and II was negatively correlated with PME activity and the total sugar (TS) content of pectin, and significantly negatively correlated with the GA content of HC I. This shows that a large amount of Al^3+^ accumulated in the cell walls, cytoplasm, and pectin at the same time, the PME activity of root tip cells was significantly enhanced, and the pectin GA and TS content was significantly increased under Al stress. However, with increased Al^3+^ concentrations in HC I and II, PME activity and the GA content of pectin decreased, and the GA content of HC I decreased significantly. There was no significant correlation between the Al^3+^ content of most subcellular components and TS in subcellular components. After Al treatment, the increased Al concentration in each subcellular fraction of the root tip had the most significant and indigenous effect on root K absorption. For example, the Al^3+^ content in cell walls, pectin, and organelles was significantly negatively correlated with K intake (*p* ≤ 0.001), but the Al^3+^ content in pectin was significantly positively correlated with P intake (*p* ≤ 0.01). In addition, PME activity was significantly correlated with K intake, and the TS content in pectin was correlated with N and P intake (*p* ≤ 0.05).

In order to further analyze and understand the response of root cell wall polysaccharide components and the PME activity of different Al-tolerant *Eucalyptus* clones to the change in Al^3+^ concentration in the root cell wall (Al^3+^(CW)), gray relational analysis (GRA) was used. The results show that the relational grades of TS (CEL), TS (PCT), and GA (HC I) were significantly higher after Al treatment. This indicates that the total sugar of cellulose and pectin and the galacturonic acid of hemicellulose I were sensitive to Al stress. They play important roles in the response of plant roots to Al toxicity (Table 1). The correlation degree of GA (HC II), PME, and GA (CEL) with Al^3+^ (CW) was always the lowest in the two clones, ranking in the bottom three, which means that the GA content of hemicellulose II and cellulose and PME activity was not sensitive to the increased Al^3+^ in the cell wall. In addition, we found that the correlation between TS (CEL), TS (PCT), and GA (PCT) and Al^3+^ (CW) in G9 was significantly different between Al-treated (Al) and control plants (CK) (*p* < 0.05), and the whole ranking order also changed. It can be inferred that G9 was more resistant to Al toxicity than G4, which may be attributed to the special role of total sugar in cellulose and pectin and GA in pectin. In contrast, no significant difference was found in the correlation degree of each appraisal item of the G4 clone among different Al treatments.

## 3. Discussion

### 3.1. Damage Degree of Root Tip Cells and Characteristics of Programmed Cell Death

The initial site of Al toxicity in plants is the root tip, especially the distal transfer zone (root tips less than 1–2 mm in length). Inhibited plant root growth is usually a direct manifestation of Al toxicity [19]. A number of studies have shown that impeded root elongation, damaged root tip morphology, and, in particular, decreased root hairs, are the first recognized symptoms of Al toxicity [4,5,28]. We also found that the root tips of G4 and G9 under Al stress displayed an enlarging basal root, and the root tips became shorter and thicker. Varying degrees of cracking occurred in the root caps and the root epidermis and its adjacent area. The reason for this may be that Al stress reduces the cellulose content of the cell wall, deforms the cells, and changes the direction of their growth from the original longitudinal growth along the root system to lateral growth, leading to expansion and thickening of the root tip base, accelerated programmed cell death (PCD), and ultimately inhibited plant root growth. Another hypothesis suggests that the decrease in cellulose synthesis leads to rapid suppression of Al-induced root elongation [29]. However, Al stress also increased the concentrations of pectin, cellulose, and hemicellulose in the root tips of the Al-sensitive *Eucalyptus* clone, and an increase in these components will reduce the mechanical ductility of the cell wall [16]. In this study, the root elongation growth of G4 was inhibited (Figure 1a–h), and the damaged morphology in the roots was more severe in G4 than in G9 (Figure 1a–h), indicating that root tip cells are more vulnerable to Al toxicity in G4 when exposed to Al stress, which may even accelerate the rate of PCD.

The degree of PCD may be negatively correlated with Al tolerance [30]. In line with our findings, Miyasaka et al. [31] reported that border cell viability in the snap bean cultivar “Dade” (Al-tolerant) was significantly higher than in the snap bean cultivar “Romano” (Al-sensitive) when these two cultivars were examined under Al stress conditions. In this experiment, the RBCs of root tips showed the highest activity in the control treatment group, followed by G9, and the RBCs in G4 showed the lowest activity and a high level of apoptosis (Figure 1i–l), indicating that the RBCs of different *Eucalyptus* clones exhibit different responses to Al stress; those that can avoid excessive Al intake have an advantage in maintaining the physiological activity and function of border cells. Other studies have shown that Al^3+^ toxicity likely limits the development of RBCs by inhibiting the activity of root cap PME [32]. Al-induced PCD mechanisms and molecular regulation pathways require further study.

### 3.2. Function of Polysaccharide Components in Root Tip Cell Wall during Al Resistance and Changes in PME Activity

Cell walls have been considered prime sites for both Al^3+^ toxicity and exclusion. We determined the distribution of Al^3+^ content to be as follows: cell wall > cytoplasm > organelles (Figure 3), which is consistent with previous reports on other woody plants (between 85 and 99.9%) [33]. Hardening of the cell wall can moderately strengthen adhesion between cells and maintain the cell shape, but excessive accumulation of Al^3+^ will aggravate the gelation degree of pectin, making the cell wall excessively hard, reducing the mechanical ductility, and destroying the structure and function of the cell wall, thereby inhibiting cell elongation and growth [5,34,35,36]. It is inferred that the extension of the cell wall is affected by the porosity and hardening of polysaccharide polymers. The major binding site of Al in cell walls is generally the polysaccharide component of the cell wall, including pectin polysaccharides, cellulose, HC I, and HC II. It mainly contains the homopolymer of GA because the negatively charged carboxylic groups have a high affinity for Al [37,38]. Accumulating evidence from some woody plants suggests that variation in pectin content and/or methylation level is correlated with Al^3+^ sensitivity or resistance. Compared to the Al-sensitive lines, all Al-resistant plants presented a higher degree of pectin methylation, supporting a positive role for pectin methylation in Al^3+^ exclusion. This implies that pectin methyl esterification and PME activity are crucial to the expression of Al toxicity and resistance. The results show that the variation in PME activity is closely related to the Al accumulation on the cell wall and is involved in the regulation of plant sensitivity to aluminum toxicity [39].

In this study, we observed that Al-sensitive G4 had more cell wall polysaccharides (Figure 4) and higher PME activity (Figure 5) in the cell walls of root tips; pectin methylation can be promoted by PME to increase cation binding sites, which will result in greater accumulation of cell wall polysaccharides and more Al binding than in Al-resistant G9. We also found that cell wall polysaccharides (GA contents) were significantly positively correlated with PME activity (*p* < 0.01) (Figure 6). This is consistent with previous reports on cereals and some woody plants, indicating that Al stress can modify the metabolism in cell walls and lead to the typical thick and rigid cell walls associated with the accumulation of polysaccharides [40,41]. The organic-acid-chelating ligands secreted by root tip cells can effectively eliminate Al or prevent it from entering the cells and alleviate the toxic symptoms [42]. Previous phases of our study showed that the concentrations of citrate and malate secreted by the root tips of the Al-resistant clone G9 increased by 50 and 25%, respectively, after being subjected to Al stress. However, there was a significant lag in the secretion of organic acids by the root tips of G4. This proves that organic acids contribute to the Al tolerance of *Eucalyptus*, and the accumulation and secretion of organic acids in cells are involved in Al detoxification [43].

### 3.3. Root Absorption Characteristics of N, P, and K Nutrients

Inhibition of plant growth by Al not only damages the microstructure and function of cell walls, but also adversely affects the absorption and utilization of nutrients by plants [44]. Most studies on crops have shown that Al toxicity strongly inhibits the quantity of crop roots and their ability to absorb and transport nutrients, which ultimately leads to nutrient deficiency or imbalance and indirectly inhibits aboveground growth [45,46,47,48]. Our results show that P uptake by the roots of two *Eucalyptus* clones increased significantly under Al stress, while Al treatment significantly inhibited N and K uptake by roots. Under Al stress, the P and K content decreased significantly in roots of Al-sensitive G4 and increased significantly in G9, and the change range was greater for G4 than G9.

The phosphorus–aluminum interaction in plants has always been a research hotspot [49]. Chen and Liao [50] found that the organic acid anions secreted by roots in acidic soil act as an effective defensive mechanism by which plants resist Al toxicity and P-deficiency symptoms. They put forward that the Al-P interaction in plants may be regulated by complex mechanisms, and the stress response of plants to Al toxicity and low P may be regulated by different signal pathways at the same time. Similar studies suggested that plants jointly regulate the combination of Al resistance and enhanced P uptake by altering root configuration and organic and non-organic acid transporters. This interaction is part of the P-stress response, suggesting that this mechanism may evolve in crop species to improve their adaptation to acidic soils [51]. Therefore, G9 had higher Al tolerance than G4 and absorbed more P, N, and K. It can be seen that G9 may have evolved comprehensive resistance to Al-P co-stress. Other studies also proved that Al stress and low P availability usually coexist in acidic soil, because the secretion of citrate in *Eucalyptus* roots enhances P availability and mobility [52].

### 3.4. Correlation of Aluminum Concentration in Root Tip Subcellular Components with Polysaccharide Content, PME Activity, and Nutrient Content

The correlation analysis in this study showed that the content of Al^3+^ held by cell walls and pectin was significantly positively correlated with the content of pectin GA and PME activity. This shows that Al induction can increase the pectin content in root tip cells. Schmohl and Horst [53] found a significant correlation between the content of pectin and Al in corn suspension cells. Other studies have shown that Al induction reduces the rate of pectin decomposition, resulting in pectin accumulation [36]. After demethylation by PME, pectin is more likely to combine with Al^3+^, Ca^2+^, Cu^2+^, etc., to form gel-like complexes, which promote cell wall hardening, thereby inhibiting the growth and development of roots. However, other studies suggested that plant species determine whether Al in cell walls is mainly accumulated in pectin, hemicellulose, or other polysaccharide components [54]. After Al treatment, the Al concentration in all the subcellular components of roots was negatively correlated with N and K absorption and positively correlated with P absorption. We believe that P uptake by the roots of both *Eucalyptus* clones was not inhibited after Al stress. On the contrary, the roots maintained a high level of P retention, which may be an important mechanism for alleviating Al toxicity and ensuring normal root function. Other studies have reported a similar conclusion that undamaged or slightly damaged “Al-resistant” roots can more effectively absorb water and nutrients in soil, especially highly unavailable water and nutrients such as P in acidic soil, suggesting that there may be multiple effects behind Al resistance and P-uptake efficiency [51].

Additionally, we used gray correlation analysis and ranking to verify the results of the correlation analysis, and studied the response of root tip cell wall polysaccharide components and PME activity of *Eucalyptus* clones G9 and G4 to Al^3+^ concentrations. The results show that, after Al treatment, compared with the control group, in the G9 clone, the total sugar content of cellulose and pectin in the root tip cell wall was significantly highly correlated with the Al^3+^ concentration. This indicates that the total sugar content in pectin and cellulose plays a key role in the Al resistance of *Eucalyptus*.

## 4. Materials and Methods

### 4.1. Plant Materials and Experimental Design

Two fast-growing 2-month-old *Eucalyptus* clones, Al-sensitive *Eucalyptus urophylla* and Al-resistant *Eucalyptus grandis* × *Eucalyptus urophylla* (G4 and G9, respectively), were provided by the Guangxi Academy of Forestry (Nanning, China), and used as test plants. The root tissues were first trimmed to 3–6 cm in length. Healthy and uniformly sized seedlings were sterilized in 1% carbendazim for 25 min and washed 3 times with sterile water. The sterilized seedlings were transferred to plastic buckets (3 seedlings in each bucket) containing 2.5 L of filter-sterilized Hoagland nutrient solution (pH 5.5). The solution was replaced with improved Hoagland’s nutrient buffer (main components: NH_4_NO_3_, KNO_3_, Ca(NO_3_)_2_, MgSO_4_, and KH_2_PO_4_, with working concentrations of NH_4_NO_3_, Ca(NO_3_)_2_, and MgSO_4_ of 80, 945, and 493 mg/L, respectively) once every 2 days. For each replacement, the improved Hoagland’s nutrient buffer (pH 5.5) was adjusted to pH 4.0 with 2 M HCl or NaOH with a gradual 0.1 decline. After 7 days, the seedlings were transferred to 0.5 mM CaCl_2_ solution (pH 4.0) to wash for 24 h. The experiment was performed in a 0.5 mM CaCl_2_ solution, and Al derived from AlCl_3_ was added (0 and 4.4 mM, pH 4.0). Treatments without Al (0 mM) served as controls. The buckets were rearranged within each block every 3 days for uniformity [55]. In our previous field study [56], the soil-soluble Al concentration of a 5-year-old plantation averaged 4.4 mM, so that concentration was used in this study. The experiments were arranged in a randomized complete design, in 4 pots containing 3 seedlings each for each treatment, with three replicates. Elongation of root tip was measured by an electronic digital vernier caliper, and root tips with a length of 3 cm were collected for measurement after 24 h.

### 4.2. Damage to the Root Tip Surface and PCD Detection

The root samples were closely laid out on double-sided conductive adhesive and observed and photographed using a field-emission scanning electron microscope (FE-SEM) (Hitachi SU-8020, Tokyo, Japan). Root tip cell apoptosis was observed using an upright fluorescence microscope (Leica DM4008, Wetzlar, Germany) under blue-light excitation. Live cells were dyed bright green under the 485 nm wavelength of fluorescent diethyl, and dead cells were stained red using propidium iodide (PI). All results were photographed using Leica application suite (LAS) (Version 3.8, Leica Microsystems, Heerbrugg, Switzerland).

### 4.3. Cell Wall Extraction in Root Tips

Tissue disruption and separation of cellular components: Subcellular component sediments in root tip cells were obtained using differential centrifugation [23]. An amount of 0.5 g of 1 cm root branch tip was cut and washed in ultrapure water. Once dried, these tissues were homogenized in cold buffer (250 mM sucrose, 50 mM Tris-HCl (pH 7.5), and 1.0 mM C_4_H_10_O_2_S_2_) and centrifuged at 3000 rpm for 15 min at 4 °C; the precipitate after centrifugation was the residue of the cell walls, which was washed 3 times with ultrapure water and dried for storage (F1). The supernatant after centrifugation was supplemented with ultrapure water until the same volume was reached and centrifuged at 15,000 rpm for 30 min at 4 °C. The precipitate obtained at the bottom was the component of organelles, which was collected as previously described (F2). The soluble supernatant obtained from the upper layer was the component of the cytoplasm (F3) [57].

Extraction of cell wall components: Five milligrams of F1, obtained via the above method, was added to 1 mL of sterile water and boiled at 100 °C for 1 h. The tissues were centrifuged at 13,200 rpm for 10 min. The supernatant was collected, and 1 mL of sterile water was added. The steps were repeated 3 times in total. The combined supernatants were collected as cell wall pectin components. The remaining pellets were collected, washed twice, freeze-dried, and resuspended in alkaline extraction buffer containing 0.1% NaBH_4_ and 4% NaOH. After 8 h, they were centrifuged at 12,000 rpm for 10 min. The supernatant was collected as hemicellulose I (HC I), and the pellets were treated 3 times. Finally, all supernatants were combined as hemicellulose II (HC II). The pellets were washed twice, freeze-dried, and labeled as cellulose.

### 4.4. PME Activity Assay and Determination of Al^3+^

To extract pectin methylesterase (PME), cell wall material (50 apices for each sample) was suspended in a 1 M NaCl solution (pH 6.0) at 4 °C for 1 h with repeated vortexing (20 s for 10 min each). The extracts were centrifuged (14,000× *g*, 10 min), and the supernatant was collected. A highly sensitive colorimetric assay method based on the condensation of an aldehyde under neutral conditions was used to analyze the cell wall PME activity. All the cell wall components obtained from the experiments, including F1, F2, F3, pectin, HC I, and HC II, were extracted in 2 M HCl for 48 h and centrifuged at 13,200 rpm for 10 min at room temperature. Then, 700 µL of supernatant was collected to determine the Al content using inductively coupled plasma emission spectrometry (ICP-AES) with an IRIS/AP optical emission spectrometer.

### 4.5. Cell Wall Polysaccharide Content and N, P, and K Content in Roots

The galacturonic acid (GA) and total sugar concentrations in each cell wall fraction were measured by using Dische colorimetry and anthrone–sulfuric acid colorimetry, respectively. Plant roots were isolated and oven-dried at 80 °C for 24 h, milled into powder, and fed through a 1 mm sieve. The content of nutrient elements (N, P, and K) in each sample was determined according to the method used by Dao [58]. N, P, and K were measured by ICP-MS (NexIon 350X, Analytik Jena AG, Jena, Germany) to find the lowest detection limits.

### 4.6. Statistical Analysis

The statistical analysis was performed using the IBM SPSS 24.0 software package (SPSS Inc., Chicago, IL, USA). One-way analysis of variance (ANOVA) and least significant difference (LSD) were performed using SPSS to test the significance of differences among treatments. Significance levels were set at *p* < 0.05 and *p* < 0.01. Gray relational analysis (GRA) was performed in SPSS to show the ranking of correlations between indicators, and the results are expressed as the mean ± standard deviation of each treatment. When using GRA, the resolution coefficient was 0.5. Microsoft Excel 2013 software (Microsoft Inc., Redmond, WA, USA) was used to create tables and bar charts. Correlation analysis (CA) was performed using Origin 2021 b (OriginLab Inc., Northampton, MA, USA), and a correlation heat map was drawn.

## 5. Conclusions

Al stress inhibited the normal division and elongation process of *Eucalyptus* root tip cells. The degree of damage to root tip cells was significantly higher in the Al-sensitive clone G4 than the Al-resistant clone G9, and the signs of programmed cell death were more obvious in G4 than G9 root tip cells. The Al^3+^ content in cell walls and pectin, as well as the content of galacturonic acid in pectin and total sugar in cellulose, was significantly higher in G4 than G9. The PME activity was higher in G4 than G9 root tips, which made it easier to catalyze pectin hydrolysis. After being subjected to Al stress, the mechanism of root absorption of N, P, and K in G9 was not as severely affected compared with that in G4, which may be an important mechanism for alleviating the common stress of high Al–low phosphorus and ensuring normal root function. An in-depth study of the accumulation, localization, and distribution of Al^3+^ in plant root tip cells was carried out to explore its effect on PME activity in cells. Along these lines, exploring methods to regulate the activity of PME is still the main research direction in the study of controlling the toxicity of Al in plants, screening highly Al-resistant plants, and addressing the trend of yield reduction caused by Al stress.

## Figures and Tables

**Table 1:** Gray correlation degree (mean ± SD) and ranking of TS, GA content, and PME activity with Al^3+^ concentration in root tip cell wall.

G9 Clone					G4 Clone				
Appraisal Items	Relational Grade (CK)	Ranking (CK)	Relational Grade (Al)	Ranking (Al)	Appraisal Items	Relational Grade (CK)	Ranking (CK)	Relational Grade (Al)	Ranking (Al)
TS(CEL)	0.828 ± 0.084 **	5	0.997 ± 0.004 **	1	TS(CEL)	0.980 ± 0.012	1	0.950 ± 0.031	1
TS(PCT)	0.996 ± 0.004 **	1	0.900 ± 0.042 **	2	TS(PCT)	0.888 ± 0.045	4	0.940 ± 0.052	2
GA(HC I)	0.867 ± 0.099	4	0.836 ± 0.074	3	GA(PCT)	0.965 ± 0.024	2	0.900 ± 0.062	3
TS(HC I)	0.671 ± 0.103	7	0.822 ± 0.082	4	GA(HC I)	0.856 ± 0.125	5	0.882 ± 0.058	4
GA(PCT)	0.949 ± 0.043 **	2	0.812 ± 0.068 **	5	TS(HC II)	0.815 ± 0.064	6	0.842 ± 0.057	5
TS(HC II)	0.911 ± 0.063	3	0.787 ± 0.161	6	TS(HC I)	0.742 ± 0.178	8	0.772 ± 0.115	6
GA(HC II)	0.704 ± 0.237	6	0.586 ± 0.123	7	GA(HC II)	0.793 ± 0.119	7	0.766 ± 0.183	7
PME	0.627 ± 0.126	8	0.501 ± 0.207	8	PME	0.895 ± 0.079	3	0.755 ± 0.160	8
GA(CEL)	0.447 ± 0.097	9	0.467 ± 0.170	9	GA(CEL)	0.468 ± 0.178	9	0.469 ± 0.179	9

Note: ** Correlation degree between Al treatment group (Al) and control group (CK), with significant difference at 0.05 level. In order to further understand the response of polysaccharide components and PME activity in root tip cell wall to Al^3+^ concentration in different Al-tolerant *Eucalyptus* clones, we used Al^3+^(CW) as the reference value to analyze the gray correlation degree of 9 appraisal items, to determine their comparative correlation and rank. A higher correlation value means a stronger correlation between the appraisal item and Al^3+^(CW), and the ranking is also higher. GA(PCT): galacturonic acid content in pectin; TS(PCT): total sugar content in pectin; GA(HC I): galacturonic acid content in hemicellulose I; TS(HC I): total sugar content in hemicellulose I; GA(HC II): galacturonic acid content in hemicellulose II; TS(HC II): total sugar content in hemicellulose II; GA(CEL): galacturonic acid content in cellulose; TS(CEL): total sugar content in cellulose. PME, pectin methylesterase activity.

## Data Availability

The data and materials will be made available on request.
